# A decade of esophageal cancer in Kazakhstan: what the national cancer registry reveals (2014–2023)

**DOI:** 10.3389/fonc.2026.1762992

**Published:** 2026-03-12

**Authors:** Altynay Beyembetova, Ruslan Akhmedullin, Diyora Abdukhakimova, Ayana Ablayeva, Aigerim Biniyazova, Oxana Shatkovskaya, Zhandos Burkitbayev, Altay Kerimkulov, Galiya Orazova, Abduzhappar Gaipov

**Affiliations:** 1 Department of Medicine, Nazarbayev University School of Medicine, Astana, Kazakhstan; 2 Board for Strategic Development, Scientific and Educational Activities, National Research Oncology Center, Astana, Kazakhstan; 3 Department of Epidemiology and Biostatistics, Astana Medical University, Astana, Kazakhstan

**Keywords:** Central Asia, DALY (disability-adjusted life years), epidemiology, esophageal cancer, Kazakhstan, population based registry, survival analysis

## Abstract

**Background:**

Esophageal cancer (EC) is a major yet understudied public health burden in Central Asia. Kazakhstan has one of the highest EC rates in the region, but national epidemiological trends and survival outcomes remain poorly reported. Epidemiological trends among younger adults are largely unexplored, despite growing concern about early-onset gastrointestinal cancers. This study assessed recent patterns in EC incidence, mortality, disease burden, and survival across Kazakhstan from 2014 to 2023.

**Methods:**

We conducted a retrospective, population-based cohort study including all EC patients registered in the National Cancer Registry between Jan 1, 2014, and Dec 31, 2023. Demographic, clinical, and survival data were extracted from registry-linked records. Early-onset EC was defined as diagnosis before age 45. Age-standardized incidence, mortality, and prevalence rates per 100,000 were calculated using the WHO World Standard Population. DALYs were estimated following GBD 2023 methodology. Survival was assessed using Kaplan–Meier methods; predictors were analyzed with Cox regression.

**Results:**

The cohort included 24,778 patients. The age-standardized incidence rate was 13.9 per 100,000 (95% CI 12.6–15.1). Mortality rose from 4.4 per 100,000 in 2014 to 10.6 per 100,000 in 2023, peaking at 12.3 per 100,000 in 2018. DALYs increased from 3,383 (95% UI 2,970–3,800) to 8,300 (95% UI 6,691–9,269) per 100,000, 95% attributable to YLL. In early-onset cases, DALYs per 100,000 tripled over ten years. Three-year survival declined from 62.9% (95% CI 59.2–66.6) at Stage I to 12.9% (95% CI 10.8–15.0) at Stage IV. Male sex (aHR 1.11), advanced stage (aHR 7.10), and alcohol disorders (aHR 1.15) were associated with poorer survival. Kyzylorda and Karaganda regions had the highest burden.

**Interpretation:**

Despite stable incidence, EC mortality in Kazakhstan has tripled over the past decade, reflecting late-stage diagnosis, limited treatment access, and persistent modifiable risk factors such as alcohol and tobacco use. These findings highlight a critical public health gap and underscore the need for early detection programs and strengthened oncology infrastructure to reduce premature mortality in Kazakhstan and neighboring countries.

## Introduction

Esophageal cancer (EC) remains one of the most lethal gastrointestinal malignancies worldwide, marked by low survival rates and frequent detection at advanced stages (1, 2). In 2024, GLOBOCAN ranked EC as the seventh leading cause of cancer-related deaths globally, with approximately 511,000 deaths annually (CR: 6.3 per 100,000 person-years) (3). The global burden of EC, measured in disability-adjusted life years (DALYs), increased by one-third between 1990 and 2021, reaching 13.2 million DALYs, 73% of which occurred in Asia (4, 5). Central Asia has remained a “blind spot” in many global cancer burden assessments, due to data gaps and underrepresentation of regional countries, which likely distorted understanding of EC patterns in this region (6).

Against this global backdrop, Kazakhstan represents a particularly underexplored setting with unique epidemiological and behavioral risk profiles. The epidemiological landscape of EC in Kazakhstan is shaped by a distinct hierarchy of modifiable risk factors. Contemporary evidence indicates that frequent consumption of fried, smoked, and heavily salted foods, together with lifestyle factors such as excessive alcohol intake, habitual consumption of very hot beverages, and heavy smoking, significantly increases the risk of upper gastrointestinal tract malignancies. (7) These behavioral risks are further compounded by molecular susceptibilities, as high-temperature meat preparation (roasting) has been linked to potential early molecular biomarkers (8).

However, despite the recognized risk environment and growing disease burden, nationally representative evidence remains limited. Prior studies were limited in scope and metrics, Igissinov et al. (2012) examined regional incidence trends using pre-2014 data, but did not assess survival, comorbidities, or DALYs, thus outdated amid healthcare reforms (9). A 2024 national analysis covered only mortality trends (2014–2022; AAPC -2.44% males, -4.03% females) and excluded data on incidence, prevalence, survival, and DALYs (10). Regional DALY estimates for Almaty (2024) highlighted local burdens yet overlooked national diversity (11).

To address these critical evidence gaps, we conducted the first comprehensive, population-based assessment of EC in Kazakhstan from 2014 to 2023. Using data from the national cancer registry, we evaluated the longitudinal dynamics of incidence, mortality, prevalence, survival, and DALYs. We further analyzed the influence of demographic, clinical, and regional factors on patient outcomes to provide a baseline for national strategies in prevention, early detection, and disease management.

## Methods

### Study design and selection criteria

This retrospective cohort study used data from the Electronic Registry of Oncology Patients (EROP), a component of the Unified Electronic Healthcare System (UNEHS), integrating all inpatient and outpatient oncology records across Kazakhstan (12). Additional information on comorbidities and missing demographic data was extracted from UNEHS (13). Each patient was registered with a unique resident identification number (RPN ID) enabling linkage across the UNEHS. The study included all first-admission EC cases recorded between Jan 1, 2014, to Dec 31, 2023. Population denominators for incidence and mortality rates, based on a total national population of approximately 20 million (as of December 2025), were obtained from the Bureau of National Statistics (14).

According to the International Classification of Diseases, Tenth Revision (ICD-10), the cohort included patients registered with codes C15.0–C15.9. In accordance with the national clinical protocol, cases coded as C16.0 (malignant neoplasm of the cardia) were also included, as cardia carcinomas may involve the distal esophagus, and primary esophageal tumors may extend into the cardia region of the stomach (15). The final cohort comprised 24,778 unique patient records (**Supplementary Figure 1**). The exclusion criteria were non-esophageal cancer diagnoses, duplicate records, invalid identifiers preventing linkage, and incomplete demographic or clinical data before 2014.

### Exposure and covariates

The dataset included information on age, sex, region, ethnicity, date of birth, date of diagnosis, and date of death, as well as ICD-10 codes for primary and secondary diagnoses, ICD-O-3 histology codes, and clinical details such as tumor stage and localization. Age at diagnosis was calculated by subtracting the date of birth from the date of diagnosis and categorized into five groups: 18–44, 45–54, 55–64, 65–74, and ≥75 years.

Ethnicity was originally recorded in more than 30 categories. We grouped the three most prevalent ethnic groups: Kazakh, Russian, and all remaining ethnicities were grouped into an “Other” category. Missing ethnicity data, which accounted for approximately 10% of records, were maintained as a separate category.

Histological subtypes were defined using the ICD-O-3 morphology classification and were divided into four major categories: squamous cell carcinoma, adenocarcinoma, other specified histological types, and unspecified tumors (**Supplementary Table 2**). Anatomical subsites were identified using ICD-10 topography codes and grouped into three anatomical thirds: upper, middle, and lower, including tumors extending into the gastric cardia (**Supplementary Table 3**). Cancer stage was classified into four categories (Stages I–IV) based on the national clinical protocol for esophageal cancer (15), which follows the AJCC TNM Cancer Staging Manual, 8th edition (2017) (16).

Data on comorbidities were extracted from inpatient and outpatient records within the UNEHS using ICD-10 coding and included hypertension, diabetes mellitus, obesity, chronic obstructive pulmonary disease (COPD), gastrointestinal disorders, and alcohol-related disorders (**Supplementary Table 4**). All comorbidity data were merged with the main EC dataset through RPN ID linkage to ensure complete patient-level information.

Geospatial analysis was conducted to visualize regional difference in prevalence using Kazakhstan’s administrative structure prior to 2018, which comprised 14 regions and two republican cities (Astana and Almaty).

### Outcomes

The primary outcomes of this study included the comprehensive assessment of incidence, prevalence, mortality, survival, and disease burden of esophageal cancer (EC) in Kazakhstan during the 10-year period from 2014 to 2023. Incidence and mortality were expressed as annual rates per 100,000 population and age-standardized to the WHO World Standard Population (16). Prevalence represented the number of individuals living with a confirmed diagnosis of EC within a given calendar year.

Two additional epidemiological indicators: mortality-to-incidence ratio (MIR) was defined as the ratio of the age-standardized mortality rate to the corresponding age-standardized incidence rate, providing an indirect estimate of survival. The proportional mortality (PM) represented the proportion of deaths attributable to EC among all EC-specific deaths during the study period, serving as a proxy for the relative contribution of EC to total cancer mortality.

The overall disease burden was quantified in DALYs, calculated as the sum of years of life lost (YLL) and years lived with disability (YLD) in accordance with the Global Burden of Disease (GBD 2023) methodology (17). YLL was derived by multiplying the number of EC-related deaths by expected remaining life years, whereas YLD was computed by multiplying disease prevalence by the respective disability weight (DW) and average disease duration. DWs were assigned as 0.288 for early-stage disease (Stages I–II), 0.451 for Stage III, and 0.540 for Stage IV. DALYs were reported per 100,000 population, overall and by age groups.

Survival outcomes were defined as the time from the date of EC diagnosis to death from any cause or the end of the observation period (December 31, 2023), whichever occurred first. Survival probabilities were estimated using the Kaplan–Meier method, and differences between groups were compared using the log-rank test. For multivariable analysis, Cox proportional hazards regression models were applied to estimate adjusted hazard ratios (aHRs) with corresponding 95% confidence intervals (CIs). Model 1 was adjusted for age, sex, and ethnicity, while Model 2 included additional adjustments for stage, histological subtype, localization, and comorbidities. The proportional hazards assumption was verified using Schoenfeld residuals and log–log survival plots.

### Statistical analysis

Data management and cleaning were conducted on a secure web server at Nazarbayev University using Stata version 16 MP2 (StataCorp, College Station, TX, USA). All analyses adhered to the Strengthening the Reporting of Observational Studies in Epidemiology (STROBE) guidelines. Demographic and clinical characteristics of patients were summarized using descriptive statistics. Continuous variables were reported as means with standard deviations (SD), whereas categorical variables were expressed as frequencies and percentages.

## Results

### Cohort characteristics

The baseline sociodemographic and medical characteristics of the study cohort are presented in **Table 1**. Between 2014 and 2023, a total of 24,778 patients were diagnosed with EC, 18,280 died (74%). Early-onset esophageal cancer accounted for 3.8% of cases (n = 951) and was characterized by a high mortality rate of 322 deaths per 1,000 person-years, despite relatively lower absolute incidence. The mean age at first EC admission was 65.4 ± 10.9 years. The vast majority of the cohort (63%) were aged 55–74 years. Men constituted 1.65 times more than females. Most patients were Kazakh (68%), followed by Russian (13%).

**Table 1:** Sociodemographic and medical characteristics of patients with esophageal cancer between 2014 and 2023.

Variable	Total n = 24,778	Alive N = 6,498	Dead n = 18,280	p-value	Mortality rate per 1,000 PY [95%CI]
Age, mean ± SD	65.4 ± 10.9	63.5 ± 10.8	66.0 ± 10.9	< 0.001	
Age groups, n (%)				< 0.001	
18-44	951 (3.8)	322 (5.0)	629 (3.4)		322 [297; 348]
45-54	2,757 (11.1)	866 (13.3)	1,891 (10.3)		325 [310; 340]
55-64	7,480 (30.2)	2,182 (33.6)	5,298 (29.0)		386 [376; 397]
65-74	8,232 (33.2)	2,130 (32.8)	6,102 (33.4)		453 [441; 464]
75+	5,358 (21.6)	998 (15.4)	4,360 (23.8)		599 [582; 617]
Sex, n (%)				< 0.001	
Female	9,351 (37.7)	2,619 (40.3)	6,732 (36.8)		400 [391; 410]
Male	15,427 (62.3)	3,879 (59.7)	11,548 (63.2)		454 [446; 462]
Ethnicity, n (%)				< 0.001	
Kazakh	16,765 (67.7)	4,549 (70.0)	12,216 (66.8)		423 [415; 430]
Russian	3,316 (13.3)	766 (11.8)	2,550 (14.0)		476 [458; 495]
Other	2,238 (9.0)	514 (7.9)	1,724 (9.4)		510 [487; 535]
Missing	2,459 (10.0)	669 (10.3)	1,790 (9.8)		388 [371; 407]
Histological subtypes, n (%)				< 0.001	
Squamous cell carcinoma	8,983 (41.8)	2,247 (40.7)	6,736 (42.2)		448 [438; 459]
Adenocarcinoma	8,579 (40.0)	2,243 (40.6)	6,336 (39.7)		463 [452; 474]
Unspecified	2,086 (9.7)	663 (12.0)	1,423 (8.9)		346 [328; 364]
Other specified	1,835 (8.5)	366 (6.6)	1,469 (9.2)		421 [400; 443]
Location subtypes, n (%)				0.001	
Upper	1,999 (8.4)	475 (7.6)	1,521 (8.7)		445 [423; 468]
Middle	5,493 (23.1)	1,379 (22.1)	4,114 (23.4)		459 [445; 473]
Lower	16,322 (68.5)	4,400 (70.3)	11,922 (67.9)		428 [421; 436]
Stages, n (%)				< 0.001	
I	1,353 (5.6)	817 (12.7)	536 (3.0)		119 [122; 145]
II	9,642 (39.7)	2,910 (45.4)	6,732 (37.7)		371 [362; 380]
III	10,103 (41.6)	2,215 (34.5)	7,888 (44.2)		500 [489; 511]
IV	3,163 (13.0)	458 (7.1)	2,705 (15.1)		800 [770; 830]

The majority of patients (69%) had tumors located in the lower third of the esophagus. Most cases were identified at advanced stages, with 40% at stage II, 42% at stage III, and 13% at the terminal stage. Squamous cell carcinoma (42%) and adenocarcinoma (40%) represented the main histological types.

Mortality reached its peak among patients aged 75 years and older, accounting for 22% of the cohort, with a rate of 599 deaths per 1,000 patient-years (95% CI: 582–617). The highest all-cause mortality rates per 1,000 patient-years occurred in men, individuals of other ethnicities, those with squamous cell carcinoma, tumors located in the middle third of the esophagus, and patients diagnosed at Stage IV.

### Incidence, prevalence and mortality

**Figure 1:**
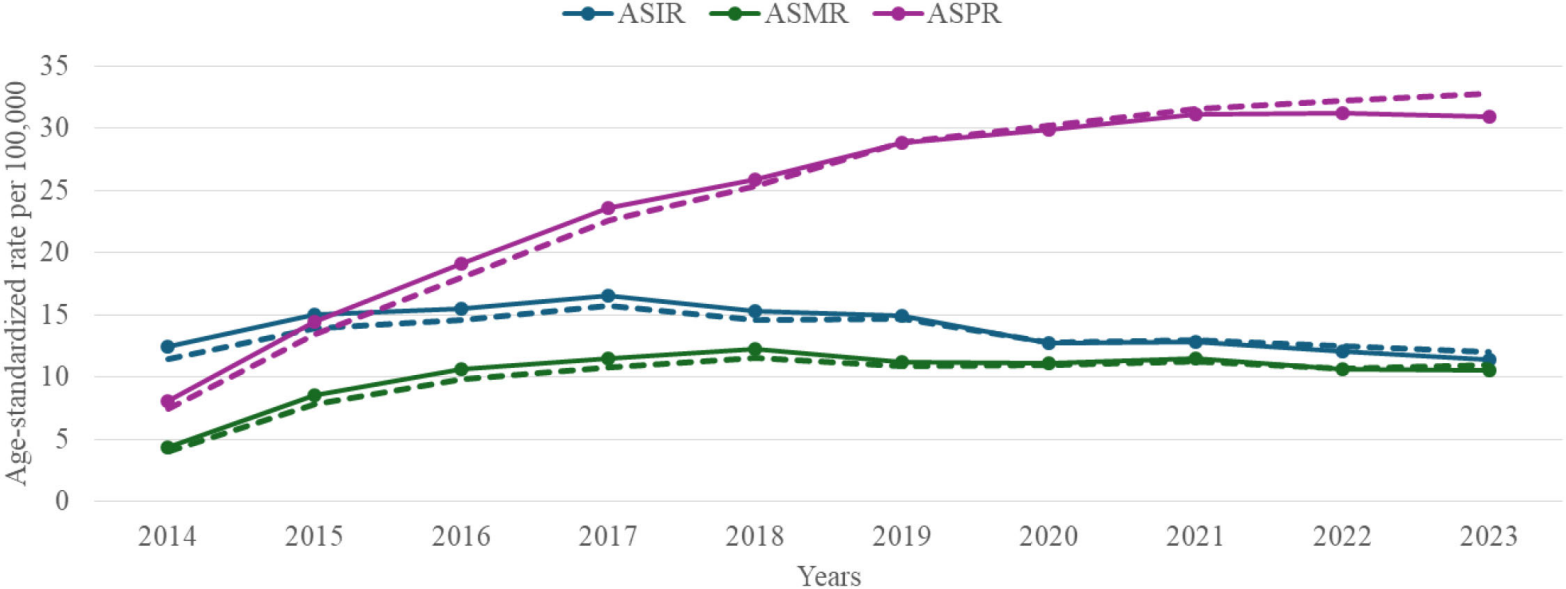
Incidence, EC-related mortality and prevalence rates in Kazakhstan per 100,000 population by year from 2014 to 2023.

EC cancer imposed a substantial and evolving burden in Kazakhstan from 2014 to 2023, with distinct patterns in age-standardized incidence (ASIR), mortality (ASMR), and proportionate mortality (ASPR) rates (**Figure 1**). Overall ASIR rose modestly from 12.5 per 100–000 in 2014 to a peak of 16.5 per 100–000 in 2017, before stabilizing at 11.4 per 100–000 by 2023. Concurrently, ASMR escalated 2.7 fold from 4.4 per 100–000 in 2014 to 12.2 per 100–000 in 2018, then declined marginally to 10.6 per 100–000 by 2023, while ASPR climbed steadily from 8.1 per 100–000 to 30.9 per 100 000.

Moreover, the mortality-to-incidence ratio (MIR) rose from 0.35 to 0.91 by 2023, while proportional mortality (PM) increased from 0.72% in 2017 to 1.69% in 2023 (**Supplementary Figure 2**).

**Figure 2:**
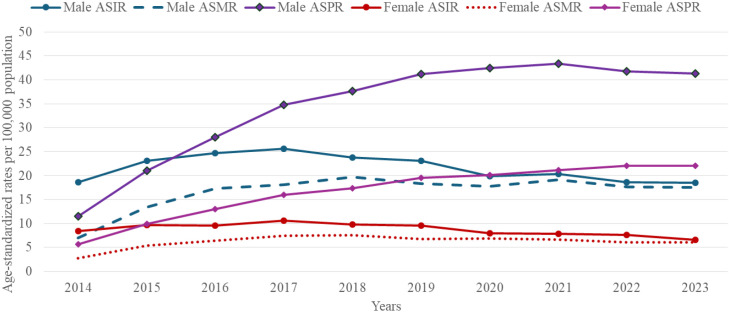
Sex-stratified incidence, EC-related mortality and prevalence rates in Kazakhstan per 100,000 population by year from 2014 to 2023.

Sex-disaggregated analysis highlighted marked male predominance across all metrics (**Figure 2**). In males, ASIR showed 38% increase from 18.5 per 100–000 population in 2014 to 25.6 in 2017, before plateauing at 18.6 by 2023; ASMR surged from 7.0 to 17.5 per 100 000 (250% increase. For females, ASIR increased from 8.5 to 10.6 per 100–000 by 2017 and then declined to 6.6 per 100–000 in 2023, whereas ASMR advanced from 2.7 to 6.0 per 100–000 by 2023. Male ASPR exceeded female levels throughout, with a mean male-to-female ASIR ratio of 2.6, consistently with sex-specific exposures in men.

### Regional variation

**Figure 3:**
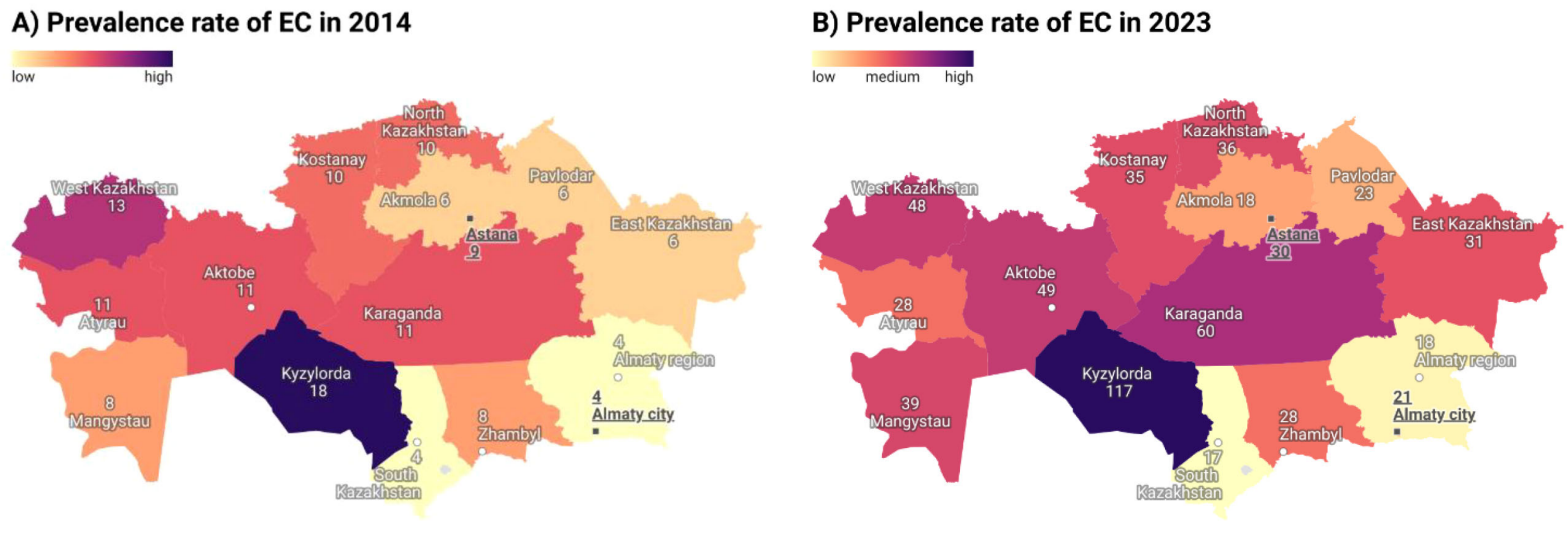
Prevalence rates of EC in Kazakhstan per 100,000 population: a) in 2014; b) in 2023.

Regional prevalence increased substantially over time. Kyzylorda and Karaganda had the highest rates in 2023 (117.3 and 59.9 per 100,000), while South Kazakhstan showed the lowest (17.1 per 100,000). Geospatial mapping revealed persistent regional clustering in central and southern regions (**Figure 3**).

### Burden of disease (DALYs)

**Figure 4:**
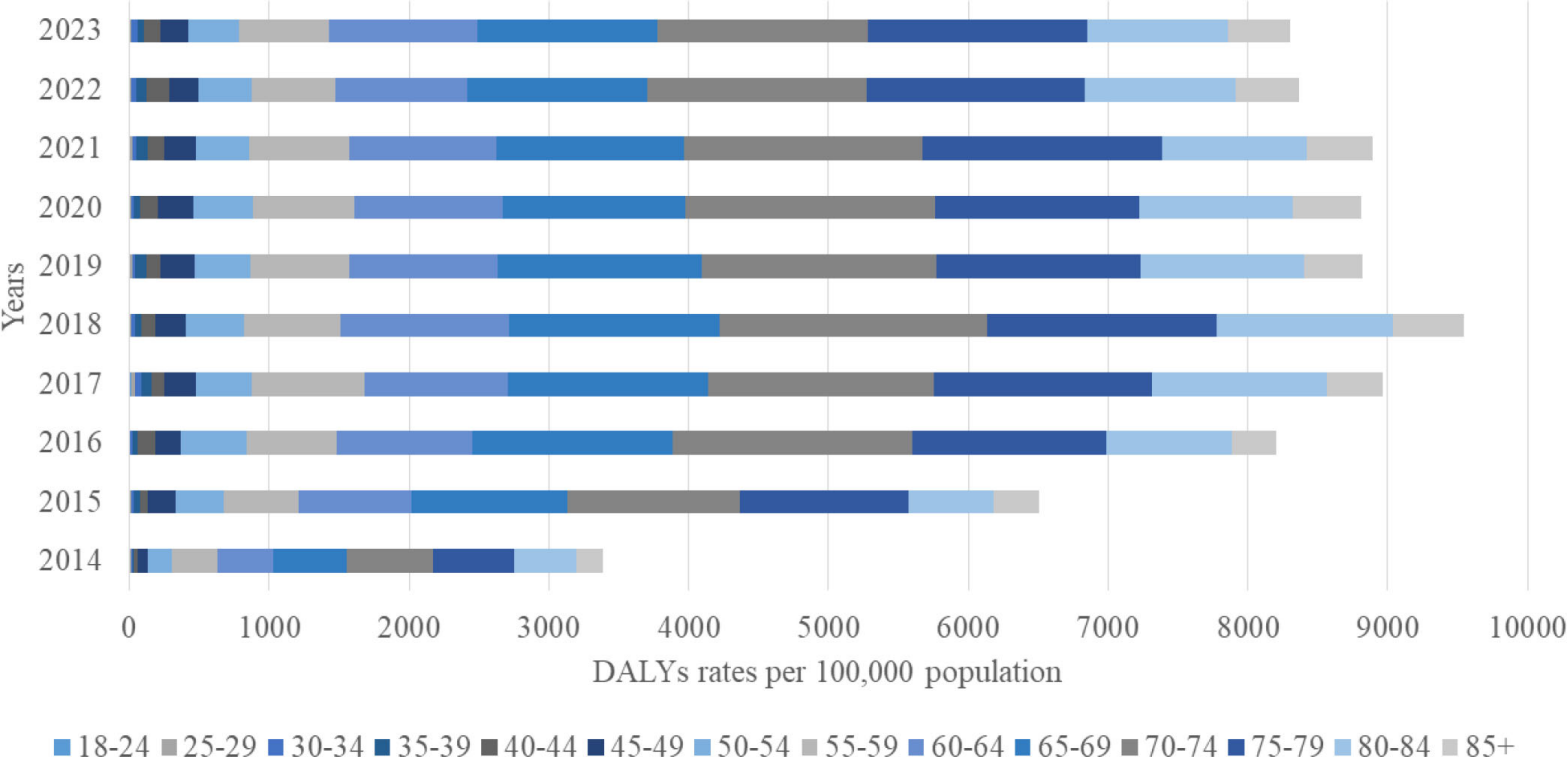
Disability-adjusted life years (DALYs) from EC per 100,000 population by age group and year, 2014–2023.

**Figure 5:**
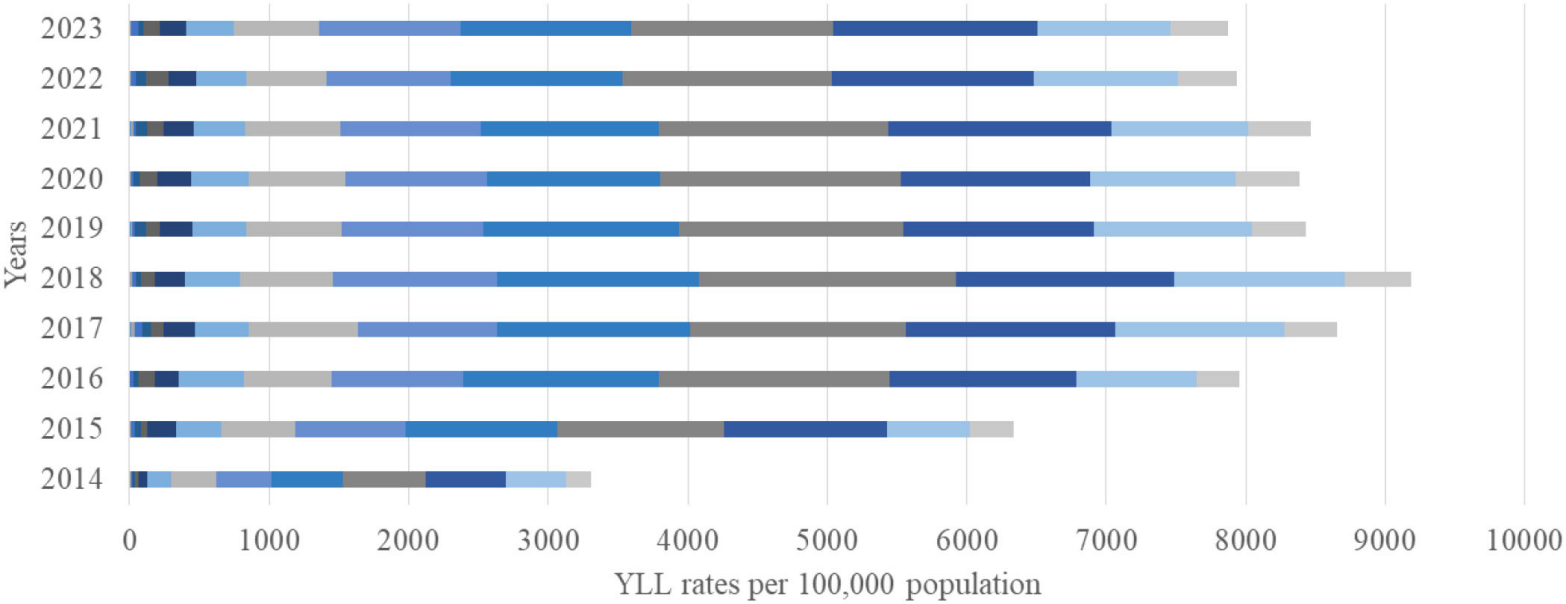
Years of life lost (YLL) from EC per 100,000 population by age group and year, 2014–2023.

**Figure 6:**
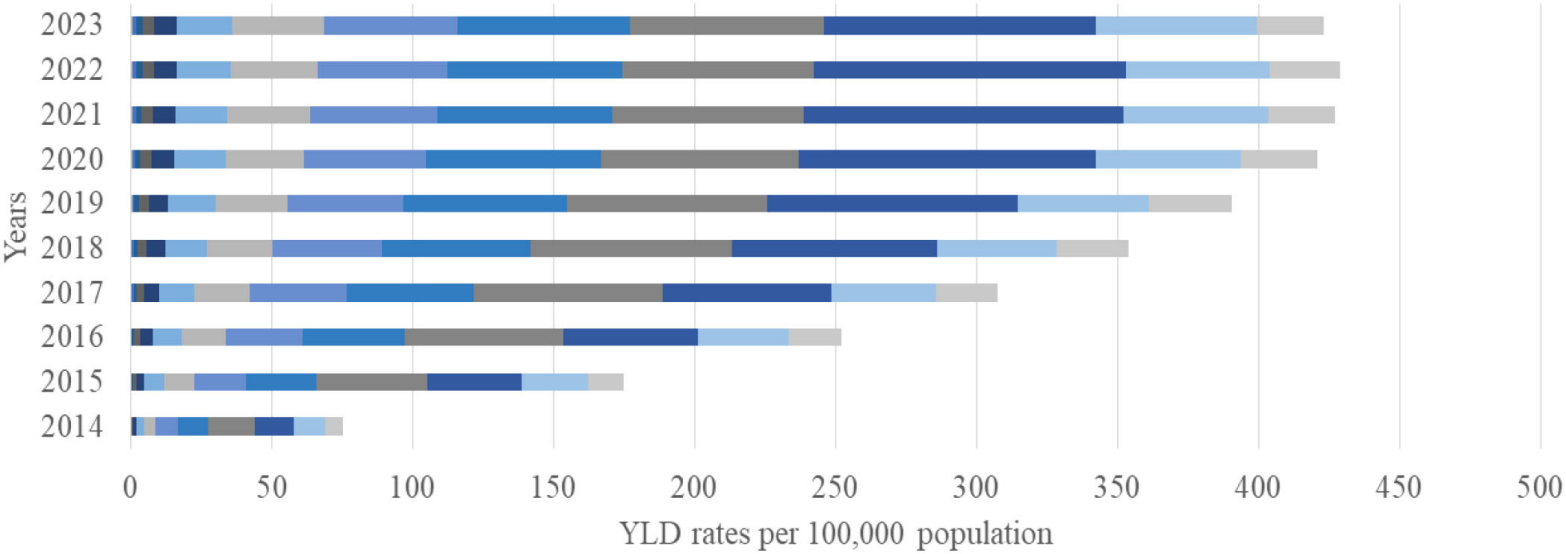
Years lived with disability (YLD) from EC per 100,000 population by age group and year, 2014–2023.

The overall DALY rate increased substantially over the study period, rising from 3,383 (95% UI 2,970–3,800) per 100,000 in 2014 to 8,300 (95% UI 6,691–9,269) per 100,000 in 2023, with YLL accounting for approximately 95% of the total burden (**Figure 4**). The greatest increase was observed among individuals aged 65–79 years, where DALYs rose from 1,723 per 100,000 (95% UI 1,560–1,880) in 2014 to 4,366 per 100,000 (95% UI 4,020–4,710) in 2023, an approximate 40% increase. YLL reached its maximum in 2018, exceeding 5055 per 100,000 (95% UI 4 800–5 310) in the 65–79-year age group (**Figure 5**). In contrast, YLD contributed with the highest levels recorded among those aged 65–79 years in 2020–238 per 100,000 (95% UI 210-266), while younger age groups (<50 years) contributed negligibly (**Figure 6**).

### Predictors of mortality

Crude and adjusted Cox proportional hazards models (**Table 2**) demonstrated that mortality risk increased markedly with advancing age, reaching its highest level among individuals aged ≥75 years (HR = 2.04; 95% CI 1.67–2.51). Male sex was independently associated with higher mortality (HR = 1.11; 95% CI 1.06–1.16), as was Russian ethnicity compared with Kazakh ethnicity (HR = 1.18; 95% CI 1.02–1.37). Patients with squamous cell carcinoma exhibited a 21% higher risk of death compared with other histological types (HR = 1.21; 95% CI 1.09–1.35). Disease stage remained the strongest prognostic factor: individuals diagnosed with Stage IV cancer had a more than sevenfold increased risk of death (HR = 7.10; 95% CI 6.22–8.12) relative to those with Stage I disease. Among comorbid conditions, alcohol-related disorders were significantly associated with increased mortality (HR = 1.15; 95% CI 1.06–1.31), whereas diabetes mellitus and gastrointestinal diseases showed modest inverse associations.

**Table 2:** Association between sociodemographic and medical parameters and all-cause mortality rates of esophageal cancer between 2014 and 2023.

Covariate	Crude HR (95% CI)	P-value	Adjusted to comorbidities HR (95% CI)	P-value
Demographics				
Age groups [<44 years old (ref)]				
45-54	1.03 [0.95; 1.13]	0.456	1.15 [0.93; 1.42]	0.439
55-64	1.12 [1.03; 1.21]	0.008	1.25 [1.02; 1.53]	0.052
65-74	1.25 [1.15; 1.35]	<0.001	1.57 [1.29; 1.92]	<0.001
≥75	1.57 [1.44; 1.70]	<0.001	2.04 [1.67; 2.51]	<0.001
Sex, [Female (ref)]				
Male	1.08 [1.05; 1.16]	<0.001	1.11 [1.06; 1.16]	<0.001
Ethnicity, [Other (ref)]				
Kazakh	1.08 [1.02; 1.14]	0.002	1.10 [0.96; 1.26]	0.172
Russian	1.25 [1.17; 1.34]	<0.001	1.18 [1.02; 1.37]	0.031
Histological subtypes, [(Other specified (ref)]				
Squamous cell carcinoma	1.14 [1.09; 1.20]	<0.001	1.21 [1.09; 1.35]	0.001
Adenocarcinoma	1.13 [1.08; 1.19]	<0.001	1.13 [1.08; 1.35]	0.027
Unspecified	0.87 [0.82; 0.92]	0.815	1.07 [0.95; 1.22]	0.275
Localization subtypes, [Upper(ref)]				
Middle	1.01 [0.96; 1.08]	0.649	0.95 [0.86; 0.99]	0.035
Lower	0.97 [0.92; 1.02]	0.253	0.92 [0.83; 1.01]	0.098
Stages, [I Stage(ref)]				
II	2.35 [2.16; 2.57]	<0.001	2.53 [2.24; 2.87]	<0.001
III	3.16 [2.90; 3.45]	<0.001	3.54 [3.13; 4.01]	<0.001
IV	5.04 [4.59; 5.53]	<0.001	7.10 [6.22; 8.12]	<0.001
Comorbidities, n(%)				
Hypertension	0.99 [0.95; 1.04]	0.706	0.96 [0.90; 1.02]	0.142
Diabetes	0.86 [0.81; 0.91]	<0.001	0.93 [0.86; 1.00]	0.052
Obesity	1.16 [0.94; 1.42]	0.161	1.13 [0.93; 1.45]	0.199
Alcohol-related disorders	1.21 [1.11; 1.32]	<0.001	1.15 [1.06; 1.31]	0.002
COPD	0.92 [0.85; 1.00]	0.051	0.95 [0.87; 1.03]	0.212
GIT diseases	0.81 [0.77; 0.85]	<0.001	0.90 [0.85; 0.96]	0.001

### Survival analysis

The overall three-year survival rate was 27.1% among women and 24.2% among men (p < 0.001). Survival outcomes varied markedly by disease stage: patients diagnosed with Stage I cancer had the most favorable prognosis, with a three-year survival of 62.9%, whereas survival declined sharply for those with Stage II, Stage III, and Stage IV disease—28.7%, 20.7%, and 12.9%, respectively (p < 0.001). Survival was marginally higher among patients with tumors located in the lower third of the esophagus (26.0%) compared with other subsites, and among individuals with unspecified histology (30.7%).

## Discussion

This study represents one of the most comprehensive national investigations to date on the epidemiology of esophageal cancer in Kazakhstan, based on population-level data from UNEHS and the EROP. Our findings reveal that while the ASIR of EC remained relatively stable between 2014 and 2023, both mortality and disease burden increased substantially over the study period. The analysis further demonstrated consistent sex-based disparities, with males exhibiting significantly higher ASIR, ASMR, and ASPR values than females across all years.

The incidence of EC in Kazakhstan is comparable to rates in neighboring Asian countries such as Turkmenistan (19.7 per 100,000), Mongolia (17.6 per 100,000), and Tajikistan (14.7 per 100,000) (18). However, when placed in a broader global context, Kazakhstan’s incidence remains significantly higher than the global average and exceeds the rates of many upper-middle-income countries, where the transition to adenocarcinoma is more pronounced (19). Within the “Asian esophageal cancer belt”, Kazakhstan occupies a middle-tier position; while its rates are lower than those reported in high-incidence provinces of China (which can exceed 30 per 100,000), the predominance of squamous cell carcinoma aligns with the East Asian epidemiological profile (20). Unlike these countries, Kazakhstan’s national cancer registry provides a more accurate and comprehensive representation of disease burden, revealing patterns that are often underestimated in global model-based estimates, including those from the Global Burden of Disease (GBD) studies, which rely heavily on extrapolated data for this region (1–3). Central Asia continues to represent a major blind spot in global cancer surveillance, and this study helps address that gap by presenting national registry-based estimates for incidence, mortality, and DALYs.

Although incidence estimates varied modestly across the decade, mortality rates surged nearly threefold, suggesting deteriorating survival outcomes. The mortality-to-incidence ratio indicated declining survival, likely due to late-stage diagnosis and limited access to timely oncologic care. While MIR serves as an indirect proxy for survival, it nonetheless reflects systemic challenges in early detection and treatment capacity (3). A temporary decline in incidence and proportional mortality during the COVID-19 pandemic likely resulted from an increase in deaths from other causes, such as respiratory and cardiovascular diseases, reducing cancer’s proportional contribution to all-cause mortality (21).

The findings confirm that men are disproportionately affected by esophageal cancer in Kazakhstan, reflecting differential exposure to major risk factors such as tobacco use, alcohol consumption, and occupational or environmental hazards. Contributing risk factors for EC incidence include family history and cultural and environmental exposure (7). Globally, the primary risk factors for SCC incidence are tobacco smoking and alcohol consumption, and their combination extremely exacerbates the risks of SCC (22). Binge drinking increases EC cancer risk three to fivefold, regardless of the type of alcohol consumed (23). Moreover, lifestyle choices such as physical inactivity, nutrition disorders, and hot beverages are established risk factors for EC (24). Obesity, Barrett’s esophagus and GERD are the main risk factors associated with AC pathology (25). In our cohort, a higher proportion of Stage II and III EC cases was observed among comorbidities presented in the study. However, conditions like Barrett’s esophagus, GERD, COPD, and obesity may be underrepresented, as undiagnosed cases could contribute to more severe disease progression and poorer prognosis compared to those censored due to competing nosology.

The incidence rise in 2016–2017 may reflect the nationwide esophagogastroduodenoscopy (EGD) screening program (2012–2018) targeting individuals aged 50–60 (26). The Ministry of Health introduced EC screening in 2012, but it was discontinued in 2018 due to the prioritization of breast, cervical, and colorectal cancer screening (27). While some studies reported a low EC detection rate (8.7% of cases), others observed an increase in early-Stage diagnoses (24.5% to 41.3%) from 2009 to 2018 (28).

The discontinuation of the national EC screening program in 2018 may have further exacerbated the high rates of late-stage diagnoses observed in our cohort. Given that over 94% of patients in our study presented with Stage II-IV disease, there is a compelling case for re-evaluating and potentially reinstating targeted endoscopic screening for high-risk groups, particularly for men over 55 in ecologically distressed regions.

The disproportionately high burden in the Kyzylorda region is likely linked to high concentrations of organochlorine pesticides and heavy metals (such as chromium and salt dust) from the dried Aral Sea bed, which have been documented in regional studies as significant environmental stressors (29, 30). Similarly, Karaganda’s high prevalence may be attributed to long-term exposure to polycyclic aromatic hydrocarbons and airborne particulate matter from coal-industrial complexes, which are established risk factors for upper aerodigestive tract cancers (31). While we could not perform a direct covariate analysis of pollutant concentrations, the geographic overlap between industrial hotspots and EC clusters suggests that environmental exposure, combined with regional disparities in screening coverage, warrants further investigation.

The growing DALY burden observed in this study provides important insights into the evolving epidemiology of EC in Kazakhstan and the broader Central Asian region. Over the ten-year period, the total DALY rate more than doubled, indicating a sharp escalation in both premature mortality and cumulative disease impact. The fact that 95% of DALYs were attributable to years of life lost (YLL) rather than years lived with disability (YLD) demonstrates that EC in Kazakhstan remains a high-fatality malignancy with very limited survivorship. This pattern is consistent with the trends observed in other low- and middle-income countries (LMICs) within the Asian esophageal cancer belt, where late presentation, diagnostic delays, and constrained access to curative therapy drive exceptionally high fatality rates (32).

Survival analysis in this study demonstrated pronounced disparities across sex, disease stage, and tumor characteristics, reflecting a consistent pattern of poor outcomes typical of settings with limited early detection capacity (4, 33). Most patients died within one to two years following diagnosis, and the overall three-year survival rate (approximately 25%) remains below both the global average and the rates reported from other upper-middle-income countries (4, 34). These findings mirror global trends in which advanced stage at diagnosis and restricted access to curative interventions, remain the primary determinants of poor survival.

Therapeutic options are further constrained by disparities in infrastructure and workforce distribution. Although surgical and radiotherapy facilities exist in major cities such as Astana and Almaty, many regional hospitals lack the capacity for minimally invasive esophagectomy, advanced radiation planning, or multimodal therapy integration. Access to palliative care and nutritional support, both critical components of EC management, inconsistent across regions. Consequently, the proportion of patients receiving curative-intent treatment is low, and adherence to international treatment guidelines remains limited.

### Strengths and limitations

This study possesses several limitations that should be acknowledged. First, the reliance on hospitalization records likely excludes pre-hospital morbidity and mortality, which may lead to underestimation of true incidence and mortality rates, particularly in rural or resource-limited regions where patients often die before reaching specialized oncology care. Such underreporting may introduce selection bias, as the study cohort predominantly represents individuals who survived long enough to receive a hospital-based diagnosis or treatment.

As the database and the registry data do not provide granular details on all non-cancer causes of death, we have addressed the reviewer’s suggestion by acknowledging that our survival analysis may be subject to competing risk bias, a common limitation in registry-based epidemiological studies. Another limitation concerns diagnostic classification; the inclusion of cases coded as C16.0 (malignant neoplasm of the cardia), in accordance with national protocols for esophagogastric junction tumors, may have led to a partial overlap with gastric cancers, influencing site-specific comparisons.

Furthermore, the registry does not systematically capture behavioral and clinical risk factors such as Barrett’s esophagus, GERD, tobacco use, and alcohol consumption, limiting our ability to assess etiologic patterns. Future research should aim to expand data linkage between cancer and outpatient registries to integrate real-world treatment data (surgery, chemotherapy, and palliative care). Such enhancements, alongside the potential reinstatement of targeted screening, are crucial for understanding survival disparities and improving access to care. Finally, given the high burden of late-stage disease identified in this study, future public health initiatives should prioritize the reinstatement of targeted endoscopic screening programs, which were previously discontinued, to bridge the gap in early detection.

## Conclusion

This first nationwide analysis of esophageal cancer (EC) in Kazakhstan reveals a substantial and escalating disease burden despite relatively stable incidence rates between 2014 and 2023. Over 94% of cases were diagnosed at Stage II or higher, with survival significantly compromised by advanced stage at diagnosis (Stage IV HR = 7.10), male sex, and alcohol-related disorders. A critical finding is the nearly threefold increase in crude mortality and the doubling of the DALY rate, where 95% of the burden is driven by premature death (YLL).

A key limitation of this study is the lack of granular data on specific surgical and chemotherapeutic interventions within the registry. To enhance the precision of future prognostic models, the national registry should: (i) integrate structured data on multimodal treatment protocols; (ii) include detailed lifestyle modules for tobacco and dietary habits; and (iii) standardize the reporting of environmental exposures in high-prevalence regions like Kyzylorda and Karaganda.

Addressing this high-fatality malignancy requires a multifaceted strategy: reinstating targeted screening for high-risk groups (men aged 55+), strengthening regional diagnostic infrastructure to reduce late-stage presentation, and ensuring equitable access to curative-intent therapies nationwide. Investment in these areas is essential to transition EC from a terminal diagnosis to a manageable condition and to reduce the disproportionate mortality burden in Central Asia.

## Data Availability

The datasets presented in this article are not readily available because in accordance with applicable regulations and ethical guidelines, we are not permitted to share any study data with third parties. This includes both raw and anonymized datasets. Requests to access the datasets should be directed to abduzhappar.gaipov@nu.edu.kz.
