# Supplementary material for: A decade of esophageal cancer in Kazakhstan: what the national cancer registry reveals (2014–2023)
Source: Front Oncol. 2026 Mar 12;16:1762992. doi: 10.3389/fonc.2026.1762992 (PMC13017348; doi:10.3389/fonc.2026.1762992)
Supplement: Supplementary file 1 [file DataSheet1.docx]

**Supplementary Materials:**

Supplementary figure F1 Esophageal cancer cohort selection processing flow-chart.


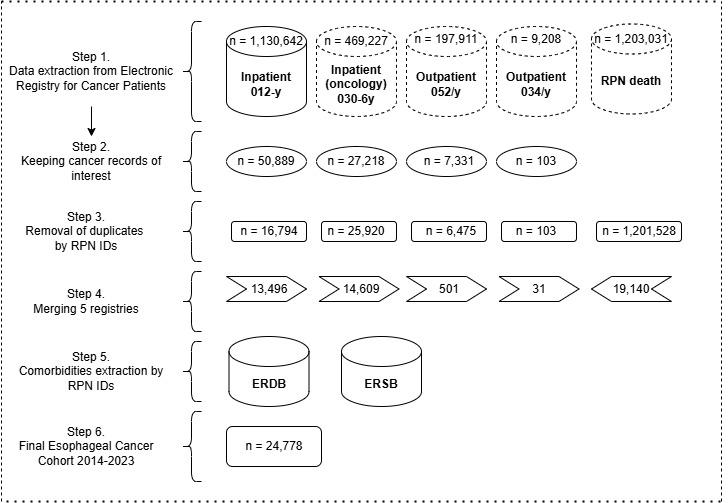


Supplementary figure F2 The chart on the mortality-to-incidence ratio (MIR) and proportional mortality (PM) over 2014-2023


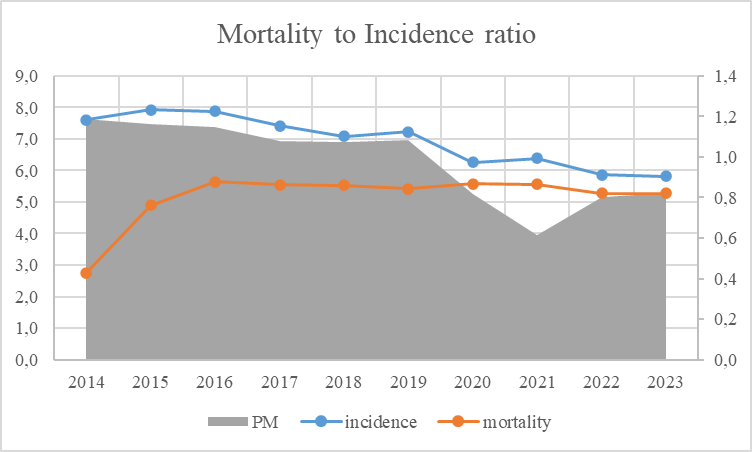


Supplementary Table S1

| EC histological subtypes | ICD-O-3 morphology codes |
| --- | --- |
| squamous cell carcinoma | 8050–8078, 8083–8084 |
| adenocarcinoma | 8140–8141, 8143–8145, 8190–8231, 8260–8263, 8310, 8401, 8480–8490, 8550–8551, 8570–8574, 8576 |
| other specified types | 8010–8035, 8800–8811, 8830, 8840–8921, 8990–8991, 9040–9044, 9120–9133, 9150, 9540–9581 |
| unspecified tumors | 8000–8005 |

Supplementary Table S2

| EC anatomical subsites | ICD-10 codes |
| --- | --- |
| upper third | C15.0, C15.1, C15.3 |
| middle third | C15.2, C15.4 |
| lower third | C15.5, C15.8–C15.9, C 16.0 |

Supplementary Table S3

| Comorbidities | ICD-10 codes |
| --- | --- |
| Hypertension | I10-13, I15, I16 |
| Diabetes | E08-10, E13 |
| Obesity | E66 |
| Alcohol related disorders | F10 |
| COPD | J44 |
| Gastrointestinal diseases | K20-23, K25-28, K29-31 |

Supplementary Table S4

| **YLL** | | | | | | | | | | |
| --- | --- | --- | --- | --- | --- | --- | --- | --- | --- | --- |
| **Age (years)** | **2014** | **2015** | **2016** | **2017** | **2018** | **2019** | **2020** | **2021** | **2022** | **2023** |
| 18–24 | 3 | 3 | 0 | 11 | 4 | 8 | 0 | 8 | 0 | 0 |
| 25–29 | 11 | 7 | 4 | 26 | 11 | 12 | 12 | 17 | 13 | 14 |
| 30–34 | 8 | 23 | 23 | 51 | 28 | 17 | 17 | 24 | 35 | 46 |
| 35–39 | 12 | 44 | 36 | 70 | 42 | 82 | 44 | 80 | 67 | 42 |
| 40–44 | 28 | 51 | 117 | 89 | 96 | 98 | 123 | 113 | 163 | 115 |
| 45–49 | 65 | 201 | 177 | 219 | 213 | 236 | 249 | 218 | 202 | 186 |
| 50–54 | 174 | 329 | 467 | 386 | 399 | 382 | 410 | 365 | 357 | 342 |
| 55–59 | 318 | 529 | 629 | 788 | 668 | 683 | 695 | 684 | 571 | 611 |
| 60–64 | 396 | 791 | 938 | 990 | 1170 | 1018 | 1017 | 1012 | 893 | 1016 |
| 65–69 | 516 | 1089 | 1399 | 1385 | 1448 | 1401 | 1240 | 1273 | 1228 | 1226 |
| 70–74 | 595 | 1192 | 1659 | 1552 | 1842 | 1607 | 1724 | 1645 | 1502 | 1441 |
| 75–79 | 571 | 1172 | 1337 | 1499 | 1569 | 1370 | 1351 | 1601 | 1448 | 1473 |
| 80–84 | 436 | 590 | 868 | 1216 | 1222 | 1130 | 1046 | 981 | 1034 | 949 |
| 85+ | 176 | 313 | 302 | 374 | 476 | 382 | 462 | 450 | 427 | 417 |
|  |  |  |  |  |  |  |  |  |  |  |
| **YLD** | **2014** | **2015** | **2016** | **2017** | **2018** | **2019** | **2020** | **2021** | **2022** | **2023** |
| 18–24 | 0 | 0 | 0 | 0 | 0 | 0 | 0 | 0 | 0 | 0 |
| 25–29 | 0 | 0 | 0 | 0 | 0 | 0 | 1 | 1 | 1 | 1 |
| 30–34 | 0 | 0 | 0 | 1 | 1 | 1 | 1 | 1 | 1 | 1 |
| 35–39 | 0 | 1 | 1 | 1 | 1 | 2 | 2 | 2 | 2 | 2 |
| 40–44 | 0 | 1 | 2 | 3 | 3 | 3 | 4 | 4 | 4 | 4 |
| 45–49 | 1 | 3 | 4 | 5 | 7 | 7 | 8 | 8 | 8 | 8 |
| 50–54 | 3 | 7 | 10 | 13 | 15 | 17 | 18 | 18 | 19 | 20 |
| 55–59 | 4 | 11 | 16 | 20 | 23 | 26 | 28 | 30 | 31 | 33 |
| 60–64 | 8 | 19 | 27 | 34 | 39 | 41 | 43 | 45 | 46 | 47 |
| 65–69 | 11 | 25 | 36 | 45 | 53 | 58 | 62 | 62 | 62 | 61 |
| 70–74 | 16 | 39 | 57 | 67 | 71 | 71 | 70 | 68 | 68 | 69 |
| 75–79 | 14 | 33 | 48 | 60 | 73 | 89 | 105 | 114 | 110 | 96 |
| 80–84 | 11 | 24 | 32 | 37 | 42 | 46 | 51 | 51 | 51 | 57 |
| 85+ | 6 | 12 | 19 | 22 | 25 | 30 | 27 | 24 | 25 | 24 |
|  |  |  |  |  |  |  |  |  |  |  |
| **DALY** | **2014** | **2015** | **2016** | **2017** | **2018** | **2019** | **2020** | **2021** | **2022** | **2023** |
| 18–24 | 3 | 3 | 0 | 11 | 4 | 8 | 0 | 9 | 0 | 0 |
| 25–29 | 11 | 7 | 4 | 26 | 12 | 12 | 13 | 18 | 14 | 15 |
| 30–34 | 8 | 24 | 23 | 51 | 29 | 18 | 18 | 25 | 36 | 48 |
| 35–39 | 13 | 45 | 37 | 72 | 44 | 84 | 45 | 82 | 69 | 44 |
| 40–44 | 28 | 52 | 119 | 91 | 99 | 102 | 127 | 117 | 167 | 119 |
| 45–49 | 66 | 204 | 181 | 225 | 219 | 243 | 257 | 226 | 210 | 193 |
| 50–54 | 176 | 336 | 477 | 399 | 414 | 399 | 429 | 383 | 376 | 362 |
| 55–59 | 322 | 540 | 645 | 807 | 692 | 709 | 723 | 713 | 602 | 644 |
| 60–64 | 404 | 809 | 965 | 1024 | 1209 | 1059 | 1060 | 1056 | 939 | 1063 |
| 65–69 | 527 | 1114 | 1435 | 1430 | 1500 | 1459 | 1302 | 1335 | 1290 | 1288 |
| 70–74 | 611 | 1231 | 1715 | 1620 | 1914 | 1678 | 1794 | 1713 | 1570 | 1510 |
| 75–79 | 586 | 1205 | 1384 | 1559 | 1641 | 1459 | 1457 | 1714 | 1558 | 1569 |
| 80–84 | 447 | 614 | 901 | 1253 | 1265 | 1177 | 1098 | 1032 | 1085 | 1006 |
| 85+ | 183 | 326 | 321 | 396 | 501 | 412 | 489 | 474 | 452 | 440 |
| **Sum** | 3383 | 6511 | 8207 | 8964 | 9542 | 8818 | 8810 | 8897 | 8369 | 8300 |
