# Supplementary material for: A decade of esophageal cancer in Kazakhstan: what the national cancer registry reveals (2014–2023)
Source: Front Oncol. 2026 Mar 12;16:1762992. doi: 10.3389/fonc.2026.1762992 (PMC13017348; doi:10.3389/fonc.2026.1762992)
Supplement: Supplementary file 2 [file Table1.docx]

# STROBE Statement—Checklist for Cohort Studies

| Item No | Recommendation | Where addressed in the manuscript |
| --- | --- | --- |
| 1(a) | Indicate the study’s design with a commonly used term in the title or the abstract | Title and Abstract: A Decade of Esophageal Cancer in Kazakhstan: What the National Cancer Registry Reveals (2014–2023) |
| 1(b) | Provide in the abstract an informative and balanced summary of what was done and what was found | Abstract (structured summary with objectives, design, results, and conclusions) |
| 2 | Explain the scientific background and rationale for the investigation being reported | Introduction, paragraphs 1–2 (context, burden of esophageal cancer, data gap in Central Asia) |
| 3 | State specific objectives, including any prespecified hypotheses | Introduction, final paragraph (objectives to assess incidence, mortality, DALYs, and survival trends) |
| 4 | Present key elements of study design early in the paper | Methods, 'Study design and population' (retrospective cohort, national registry data, 2014–2023) |
| 5 | Describe the setting, locations, and relevant dates, including periods of recruitment, exposure, follow-up, and data collection | Methods, 'Study design and population' (Kazakhstan, 2014–2023, follow-up to December 31, 2023) |
| 6(a) | Give the eligibility criteria, and the sources and methods of selection of participants. Describe methods of follow-up | Methods, 'Case definition and data sources' (ICD-10 codes, inclusion/exclusion criteria, linkage via RPN IDs) |
| 6(b) | For matched studies, give matching criteria and number of exposed and unexposed | Not applicable (unmatched cohort study) |
| 7 | Clearly define all outcomes, exposures, predictors, potential confounders, and effect modifiers. Give diagnostic criteria, if applicable | Methods, 'Exposure and covariates' and 'Outcomes' (definitions, ICD codes, stage, histology, comorbidities) |
| 8 | For each variable of interest, give sources of data and details of methods of assessment (measurement). Describe comparability of assessment methods if there is more than one group | Methods, 'Data sources' (UNEHS, Population Registry, WHO, Bureau of National Statistics, NSDI) |
| 9 | Describe any efforts to address potential sources of bias | Methods, 'Exposure and covariates' (handling of missing ethnicity data, inclusion as separate category) |
| 10 | Explain how the study size was arrived at | Methods, 'Case definition and data sources' (flow diagram, inclusion/exclusion, 24,778 final cases) |
| 11 | Explain how quantitative variables were handled in the analyses. If applicable, describe which groupings were chosen and why | Methods, 'Exposure and covariates' (age categorized into five groups, stage I–IV, regional aggregation) |
| 12(a) | Describe all statistical methods, including those used to control for confounding | Methods, 'Statistical analysis' (Cox models, adjustment for sociodemographic and clinical covariates) |
| 12(b) | Describe any methods used to examine subgroups and interactions | Methods, 'Statistical analysis' (stratification by sex, age, stage, region, and histology) |
| 12(c) | Explain how missing data were addressed | Methods, 'Statistical analysis' (missing data <10% treated as separate category) |
| 12(d) | If applicable, explain how loss to follow-up was addressed | Methods, 'Statistical analysis' (censoring at last follow-up or death from other causes) |
| 12(e) | Describe any sensitivity analyses | Not performed |
| 13(a) | Report numbers of individuals at each stage of study—e.g., numbers potentially eligible, examined for eligibility, confirmed eligible, included in the study, completing follow-up, and analysed | Results, Figure S1 (flow diagram of case selection) |
| 13(b) | Give reasons for non-participation at each stage | Figure S1 (exclusions due to missing or duplicate records) |
| 13(c) | Consider use of a flow diagram | Figure S1 (flow diagram included) |
| 14(a) | Give characteristics of study participants (e.g., demographic, clinical, social) and information on exposures and potential confounders | Results, Table 1 (baseline characteristics by sex and stage) |
| 14(b) | Indicate number of participants with missing data for each variable of interest | Results, Table 1 (ethnicity missing 10%) |
| 14(c) | Summarise follow-up time (e.g., average and total amount) | Methods, 'Statistical analysis' (follow-up period 2014–2023; censoring described) |
| 15 | Report numbers of outcome events or summary measures over time | Results, Tables 2–4 (incidence, mortality, prevalence, DALYs) |
| 16(a) | Give unadjusted estimates and, if applicable, confounder-adjusted estimates and their precision (e.g., 95% confidence interval). Make clear which confounders were adjusted for and why they were included | Results, Table 5 (Cox regression results: Model 1 sociodemographic, Model 2 clinical adjustments) |
| 16(b) | Report category boundaries when continuous variables were categorized | Methods, 'Exposure and covariates' (age and stage categories) |
| 16(c) | If relevant, consider translating estimates of relative risk into absolute risk for a meaningful time period | Not applicable |
| 17 | Report other analyses done—e.g., analyses of subgroups and interactions, and sensitivity analyses | Results, Supplementary Tables S3–S5 (stratified results) |
| 18 | Summarise key results with reference to study objectives | Discussion, opening paragraph |
| 19 | Discuss limitations of the study, taking into account sources of potential bias or imprecision. Discuss both direction and magnitude of any potential bias | Discussion, final paragraph (limitations: underreporting, registry completeness, missing data) |
| 20 | Give a cautious overall interpretation of results considering objectives, limitations, multiplicity of analyses, results from similar studies, and other relevant evidence | Discussion, paragraphs 2–4 |
| 21 | Discuss the generalisability (external validity) of the study results | Discussion, concluding paragraph |
| 22 | Give the source of funding and the role of the funders for the present study and, if applicable, for the original study on which the present article is based | Funding statement and Acknowledgements |
